# ELIO CONSENTINO: 04/16/1937 - 08/02/2024

**DOI:** 10.1590/1413-785220243203e289310i

**Published:** 2024-08-26

**Authors:** Olavo Pires de Camargo, Reynaldo Jesus-Garcia, Pedro Péricles Ribeiro Baptista, Alex Guedes, Suely Akiko Nakagawa, Eduardo Sadao Yonamine

**Affiliations:** 1. Instituto de Ortopedia e Traumatologia, Hospital das Clínicas, Universidade de São Paulo, São Paulo, SP, Brazil.; 2. Department of Orthopedics and Traumatology, Escola Paulista de Medicina, Universidade Federal de São Paulo, São Paulo, SP, Brazil.; 3. Instituto Arnaldo Vieira de Carvalho, São Paulo, São Paulo, SP, Brazil.; 4. Department of Experimental Surgery and Surgical Specialties, Faculdade de Medicina da Bahia, Universidade Federal da Bahia, Salvador, BA, Brazil.; 5. Centro de Referência de Sarcomas e Tumores Ósseos, AC Camargo Cancer Center São Paulo, SP, Brazil.; 6. Faculdade de Ciências Médicas da Santa Casa de São Paulo, São Paulo, SP, Brazil.

**Figure:**
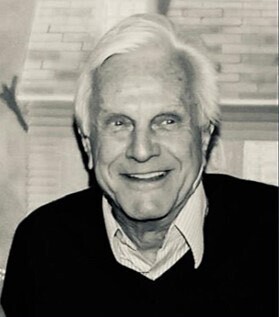
Elio Consentino

Dr. Elio Consentino, one of the pioneers of Orthopedic Oncology in our country and a mentor to a generation of orthopedists in Brazil and Latin America, passed away on August 2.

Graduated in 1963 from the Paulista School of Medicine, he began his medical residency in 1964 in the Department of Orthopedics and Traumatology at Santa Casa de São Paulo (Fernandinho Simonsen Pavilion),^1^ at the time directed by Professor Domingos Define.^2^ The Pavilion had weekly meetings, always held on Wednesdays, with an entire hour dedicated to the presentation and discussion of bone tumor cases. The memorable meetings, conducted by Professor José Donato de Próspero (pathologist) and Dr. Bartolomeu Bartolomei (orthopedist), ended up arousing the interest of several orthopedists, especially Dr. Elio Consentino, who became enthusiastic about the topic, remaining at the Institution after the end of his residency.^2^

In 1969, Professor José Soares Hungria Filho was appointed Director of the Pavilion. When remodeling the Department of Orthopedics and Traumatology, he divided it into subspecialty groups, including Orthopedic Oncology, appointing Dr. Elio as its head. Since then, the group has developed, always attached to the Pathology Service led by Professor Donato.^2^ All cases treated in the Pavilion were cataloged, generating a considerable archive of bone pathology,^2^ led to numerous invitations to Dr. Elio for lectures and case presentations at conferences, symposiums, and workshops, among other events.

In 1974, Professor Flávio Pires de Camargo invited the Argentine doctor Dr. Roberto Fabroni to an event at IOT-HC-FMUSP to present the results of the use of custom-made unconventional prostheses made of polyethylene, which he developed in his country. Dr. Elio attended the event and was impressed with the results presented. From the initial contact with Dr. Fabroni, the opportunity arose for a one-month visit to Argentina, which he made together with Professor Roberto Attilio Lima Santin, to get to know the implant factory, attend surgeries, and follow the postoperative period of patients undergoing resection procedures with replacement with endoprostheses. Since 1975, Dr. Elio started performing these operations in Brazil, with prostheses imported from Argentina, until, in 1977, a factory was established in São Paulo.^3^

The growth and dissemination of the group attracted doctors from Brazil and Latin America interested in specific training in Orthopedic Oncology at the Fernandinho Simonsen Pavilion. Dr. Elio “wrote with a scalpel” – his solutions for treating musculoskeletal tumors, always very original and advanced for the time, were carried forward more by “live” transmission than by publications in journals or books.^4^

In August 1988, during the Brazilian Congress of Orthopedics and Traumatology, held in Brasília-DF, the Committee of Musculoskeletal Tumors of the Brazilian Society of Orthopedics and Traumatology was created, with Dr. Elio as one of the founders and first President.^5^,^6^

In 1995, Dr. Elio and Dr. Fabroni conceived the creation of the *Latin American Society of Musculoskeletal Tumors* (SLATME).^3^ In early 1996, visits were made to Latin American countries seeking to invite professionals interested in the subspecialty to join this society, culminating in the realization of the Conference on Updates in Bone and Soft Tissue Tumors, in Guarujá, in October 1996, where the proposal was endorsed and Dr. Elio was appointed as the first President.^3^,^4^

Under the guidance of Dr. Elio, an ethical and prepared individual who took pleasure in teaching, an entire first generation of masters in Orthopedic Oncology was created, professionals who passed on this knowledge to subsequent generations, contributing to the development of this subspecialty in Brazil and Latin America.

Dr. Elio leaves behind his wife, Eliane, four sons (Luciano, Adriana, Fabiana, and Marcelo), and six grandchildren (Fernanda, Camila, Paulo, Felipe, Olivia, and Theo).
